# High Concentration of Melatonin Regulates Leaf Development by Suppressing Cell Proliferation and Endoreduplication in *Arabidopsis*

**DOI:** 10.3390/ijms18050991

**Published:** 2017-05-05

**Authors:** Qiannan Wang, Bang An, Haitao Shi, Hongli Luo, Chaozu He

**Affiliations:** Hainan Key Laboratory for Sustainable Utilization of Tropical Bioresources and College of Biology, Institute of Tropical Agriculture and Forestry, Hainan University, Haikou 570228, China; wangqiannan@hainu.edu.cn (Q.W.); anbang@hainu.edu.cn (B.A.)

**Keywords:** melatonin, leaf growth, cell proliferation, cell expansion, endoreduplication, *Arabidopsis*

## Abstract

*N*-acetyl-5-methoxytryptamine (Melatonin), as a crucial messenger in plants, functions in adjusting biological rhythms, stress tolerance, plant growth and development. Several studies have shown the retardation effect of exogenous melatonin treatment on plant growth and development. However, the in vivo role of melatonin in regulating plant leaf growth and the underlying mechanism are still unclear. In this study, we found that high concentration of melatonin suppressed leaf growth in *Arabidopsis* by reducing both cell size and cell number. Further kinetic analysis of the fifth leaves showed that melatonin remarkably inhibited cell division rate. Additionally, flow cytometic analysis indicated that melatonin negatively regulated endoreduplication during leaf development. Consistently, the expression analysis revealed that melatonin regulated the transcriptional levels of key genes of cell cycle and ribosome. Taken together, this study suggests that high concentration of melatonin negatively regulated the leaf growth and development in *Arabidopsis*, through modulation of endoreduplication and the transcripts of cell cycle and ribosomal key genes.

## 1. Introduction

*N*-acetyl-5-methoxytryptamine (Melatonin), as an endogenous biomolecule first discovered in the pineal gland of cow [1], was later found to be extensively in almost all living organisms, including plants [2,3,4,5,6]. Melatonin participates in the regulation of many physiological processes in animals, including sleep, circadian rhythms, body temperature regulation, immune responses, etc. [7,8,9,10]. In the most recent 20 years, studies on melatonin have revealed that it functions as an important messenger in regulating the response of plant to both abiotic and biotic stresses, including cold, heat, salt, drought, cadmium, zinc sulfate, and pathogen attacks [11,12,13,14,15,16,17,18,19,20,21,22]. Melatonin also participates in the processes of root growth and architecture, shoot development, flowering, seed germination and fruit ripening [23,24,25,26,27,28,29]. In addition, the auxin-like effects of melatonin are controversial [23,30,31,32,33,34,35,36,37].

Organ size is an important feature of plant morphology. With the determinate growth fate, plant organ size is relative constancy within a given species; however, it varied remarkably among different plant species, indicating the final size of an organ is mainly determined by endogenous generated signals [38,39]. Besides, the motionlessness of plants makes them more susceptible to exogenously generated signals, such as light, temperature, water, nutrients, plant hormones, biotic stress, and so on [40,41,42]. The leaf occurrence and development in *Arabidopsis thaliana* consists of three important processes: leaf primordia initiation, leaf polarity establishment and leaf growth [43]. Cell proliferation and expansion are two different but interconnected events that are necessary for the process of leaf growth. Cell proliferation generates new cells from primordium with relatively small cell size, while cell expansion causes further growth in size of the new cells [44,45]. During the switching phase, cell proliferation of leaves first stops at leaf-apex, thereafter most cells exit the cell cycle gradually, and then the leaf cells start to expand from the tip to the base [45,46,47]. The final leaf size is the result of strict spatial and temporal genetic control and coordination of these two successive but overlapping phases [45]. However, only changes on level of cell proliferation or cell expansion will not certainly alter the leaf size because of the compensation effect caused by reduced cell proliferation and the DNA ploidy increase resulted from endoreduplication [38,48]. Thus, leaf size is not simply controlled by cell number or cell size [38,48,49].

To date, although numerous studies have shown that melatonin play important roles in plant growth, whether melatonin participates in regulating leaf growth and the underlying mechanism are still unclear in higher plants. In this study, wild-type *Arabidopsis* (WT, Columbia-0 ecotype) were treated with various concentrations of melatonin, and the results showed that high concentration of melatonin dramatically suppressed leaf growth by reducing the cell number and cell size. Further comprehensive analyses suggested that melatonin might regulate the leaf growth by inhibiting cell proliferation and endoreduplication.

## 2. Results

### 2.1. High Concentration of Melatonin Suppresses the Leaf Growth in Arabidopsis by Reduced Cell Size and Cell Number

**Figure 1:**
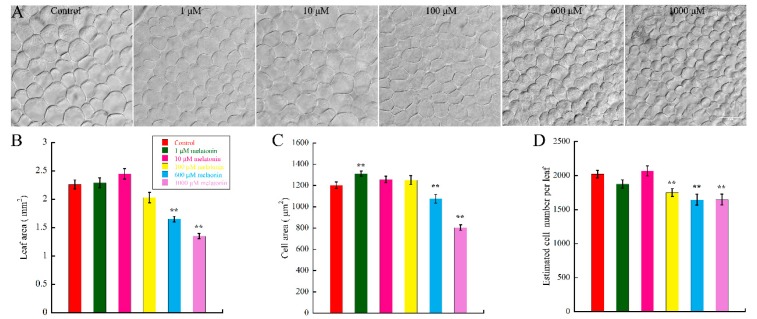
Effect of high concentration of melatonin on leaf size, cell size and cell number of *Arabidopsis* cotyledons. After one-day germination, seeds were transferred to 1/2 MS (Murashige & Skoog) medium with indicated concentrations of melatonin for another six days, and the leaf area, cell area and cell number were measured with software Image J. (**A**) Palisade cells of cotyledons cultured in control and melatonin treated medium. Scale bar = 50 μm; (**B**) Leaf area; (**C**) Cell area; and (**D**) Estimated cell number per leaf of *Arabidop*sis growing on medium with control and increasing concentration of melatonin. More than 20 cotyledons from 10 seedlings per experiment from three independent experiments were measured for statistic analysis. Values represent mean ± SD, ** *p* < 0.01 by a Student’s *t*-test.

To investigate the effect of melatonin on leaf growth in *Arabidopsis*, WT (Col-0) seeds were firstly germinated for one day on 1/2 MS (Murashige & Skoog) media to alleviate the effect of melatonin on seeds germination; then, the germinated seeds were transferred onto new 1/2 MS media with different concentrations of melatonin and cultured for another six days. Cotyledons of the samples were harvested and photographed at indicated time-points. By measuring and statistical analysis, results showed that the melatonin treatment decreased the leaf size in concentration-dependent manner (Figure 1B). After six-day treatment, both 600 and 1000 μM melatonin caused significant decrease of leaf size, with the inhibition level of 27.4% and 40.3%, respectively. The regulation of organ size is mediated by cell proliferation and cell expansion [50]. Many regulatory factors eventually lead to changes in cell number or cell size. Thus, we wonder if melatonin reduces the leaf size by affecting cell size or cell number of cotyledons. To test our hypothesis, cotyledons of seedlings grown under indicated concentrations of melatonin were then bleached and cleared for observation. We found that both average cell area and cell number of palisade cells were significantly suppressed in cotyledons treated with 600 and 1000 μM melatonin (Figure 1A,C,D). Our data showed that low concentration of melatonin had little effect on cotyledon growth (Figure 1), and our previous study indicated that 10–50 μM melatonin had little effect on endogenous melatonin content [18]. Thus, 1000 μM melatonin was used to treat the *Arabidopsis* seedlings for further analyses in this study.

**Figure 2:**
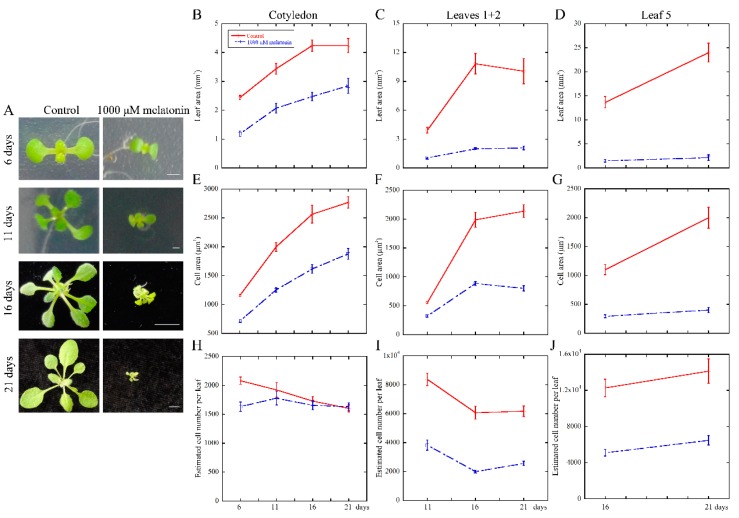
High concentration of melatonin restricted the development of leaf area by reducing the cell area and cell number of *Arabidopsis* seedlings. One-day-old seeds were kept growing under control and 1000 μM melatonin for another 6, 11, 16 and 21 days. (**A**) Digital images of control and 1000 μM melatonin treated *Arabidopsis* seedlings at a series of distinct developmental stages. Scale bars represented 1, 1, 5 and 5 mm, respectively, from the top to bottom; (**B**–**J**) Growth kinetic analysis of leaves in *Arabidopsis* seedlings grown under control and 1000 μM melatonin. (**B**) Leaf are; (**E**) cell area and (**H**) estimated cell number of cotyledons selected at 6, 11, 16 and 21 days after germination; (**C**) Leaf area; (**F**) cell area and (**I**) estimated cell number of leaves 1 + 2 harvested at 11, 16 and 21 days, and (**D**) Leaf area; (**G**) cell area and (**J**) estimated cell number of the fifth leaves were picked at 16 and 21 days were analyzed. At least 10 seedlings were selected to measure the leaf area, cell area and estimated cell number per leaf. Three independent experiments were measured for statistical analysis. Values represent mean ± SE.

To deeply reveal the long-time effect of melatonin on the growth and development of *Arabidopsis* leaves, we performed the kinetic analysis after treatment at 6, 11, 16 and 21 days. The results showed that the treated seedlings exhibited smaller size compared with the control (Figure 2A). Thus, the cotyledons at 6, 11, 16 and 21 days; first pair of true leaves (leaves 1 + 2) at 11, 16 and 21 days; and the fifth leaves (leaf 5) at 16 and 21 days were selected for statistical analysis. We found that the leaf area was inhibited dramatically by melatonin treatment (Figure 2B–D). Both palisade cell area and cell number per leaf were decreased in the melatonin treated plants, except for the palisade cell number in cotyledons harvested at 11, 16 and 21 days (Figure 2E–J). Taken together, these results indicated that melatonin-mediated repression of leaf size might be due to both reduced cell size and cell number per leaf, as evidenced by the inhibition of cell proliferation and expansion during leaf growth in *Arabidopsis*.

### 2.2. Melatonin Negatively Affects the Cell Division Rate of Leaves During Leaf Growth

**Figure 3:**
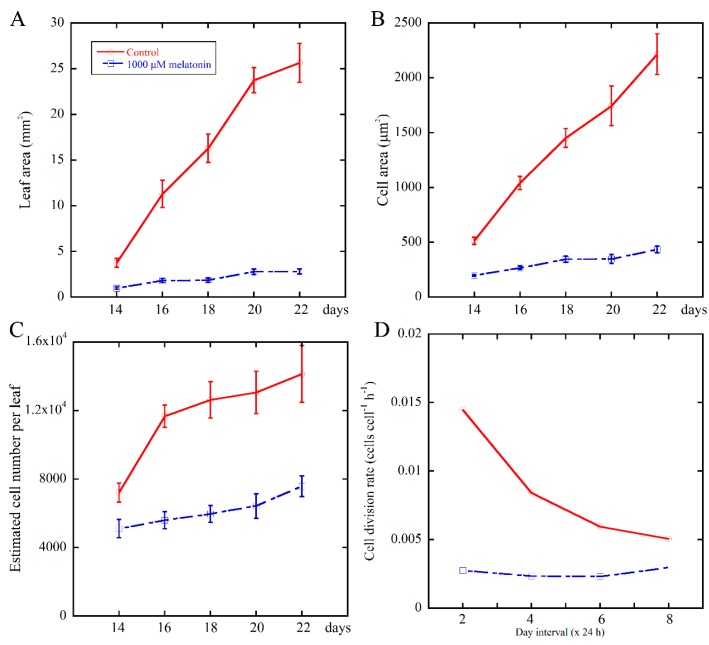
Growth kinetics of the fifth leaves in *Arabidopsis* seedlings grown under control and 1000 μM melatonin. Leaf area (**A**); cell area (**B**); estimated cell number per leaf (**C**); and cell division rate (**D**) were determined from at least 10 leaves for each independent experiments. Three biological replicates were taken for statistical analysis. Values represent mean ± SE.

To further illustrate the relationship between melatonin and cell proliferation during leaf development, we continued monitoring the growth kinetics of the fifth leaves of *Arabidopsis* after treatment for 14 to 22 days. Then, the cell division rate of palisade cells were estimated [51], as shown in Figure 3B,C. We found that 1000 μM melatonin treatment obviously reduced the cell division rate of palisade cells (Figure 3D), suggesting that melatonin treatment caused defective cell proliferation. In the 48-h interval between Day 14 and Day 16, the cell division rate of control and melatonin treated palisade cells was 0.0145 cells cell^−1^ h^−1^ and 0.0028 cells cell^−1^ h^−1^, respectively. The cell cycle duration of control palisade cells was 1/0.0145 h (68.9 h), while that of the melatonin treated samples was 1/0.0028 h (357.1 h), indicating that the duration of the cell cycle was prolonged by the melatonin treatment. These data suggested that melatonin suppressed the cell number in *Arabidopsis* leaves by reducing the cell division rate.

### 2.3. Melatonin Regulate Endoreduplication during Leaf Development

**Figure 4:**
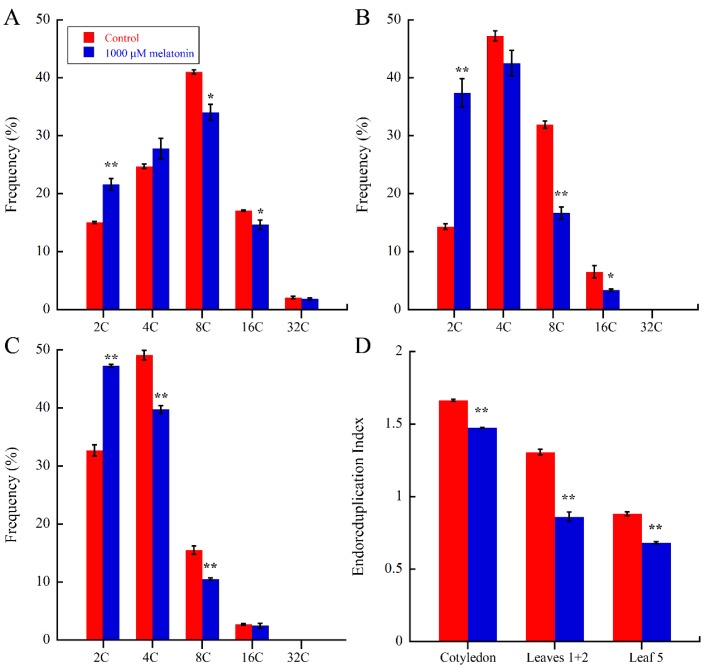
Nuclear polyploidization analysis of leaves in control and 1000 μM melatonin treated *Arabidopsis* seedlings at 21 days after one-day germination. Ploidy levels of cotyledons (**A**); leaves 1 + 2 (**B**); and the fifth leaves (**C**) were performed by flow cytometic analysis. Three biological replicates were taken for statistical analysis. Values represent mean ± SD, * *p* < 0.05; ** *p* < 0.01 by a Student’s *t*-test and (**D**) endoreduplication index (EI) analyzed from flow cytometic data of cotyledons, leaves 1 + 2 and the fifth leaves. EI represents the average number of endocycles undergone by a typical nucleus and was calculated from these percentage values as follows: EI = [(0 × 2C) + (1 × 4C) + (2 × 8C) + (3 × 16C) + (4 × 32C)]/100 [55]. Values represent mean ± SD, ** *p* < 0.01 by a Student’s *t*-test.

A large increase of cell size is mainly achieved by cell expansion, which is closely related with endoreduplication, in which several rounds of DNA replication take place [52,53,54]. The above results suggested that melatonin negatively altered the cell size of *Arabidopsis*, so we wondered that whether melatonin actually affects DNA ploidy during cell cycle. The cotyledons, leaves 1 + 2 and the fifth leaves in *Arabidopsis* 21-day seedlings of control and melatonin treated were harvested for flow cytometic analysis. As shown in Figure 4A–C, melatonin caused significant increase of 2C fractions in all three tissues, and corresponding decrease of 8C and 16C fractions in cotyledons and leaves 1 + 2, while the 4C and 8C fractions of the fifth leaves were reduced by melatonin. The endoreduplication index (EI) represents the average number of endocycles performed per cells. As showed in Figure 4D, melatonin significantly reduced the EI values of cotyledons, leaves 1 + 2 and the fifth leaves by 11.42%, 34.2% and 22.7%, respectively. These results showed that melatonin caused reduced DNA content and EI during leaf development.

### 2.4. Melatonin Regulated Several Cell Cycle and Ribosomal Related Genes

**Figure 5:**
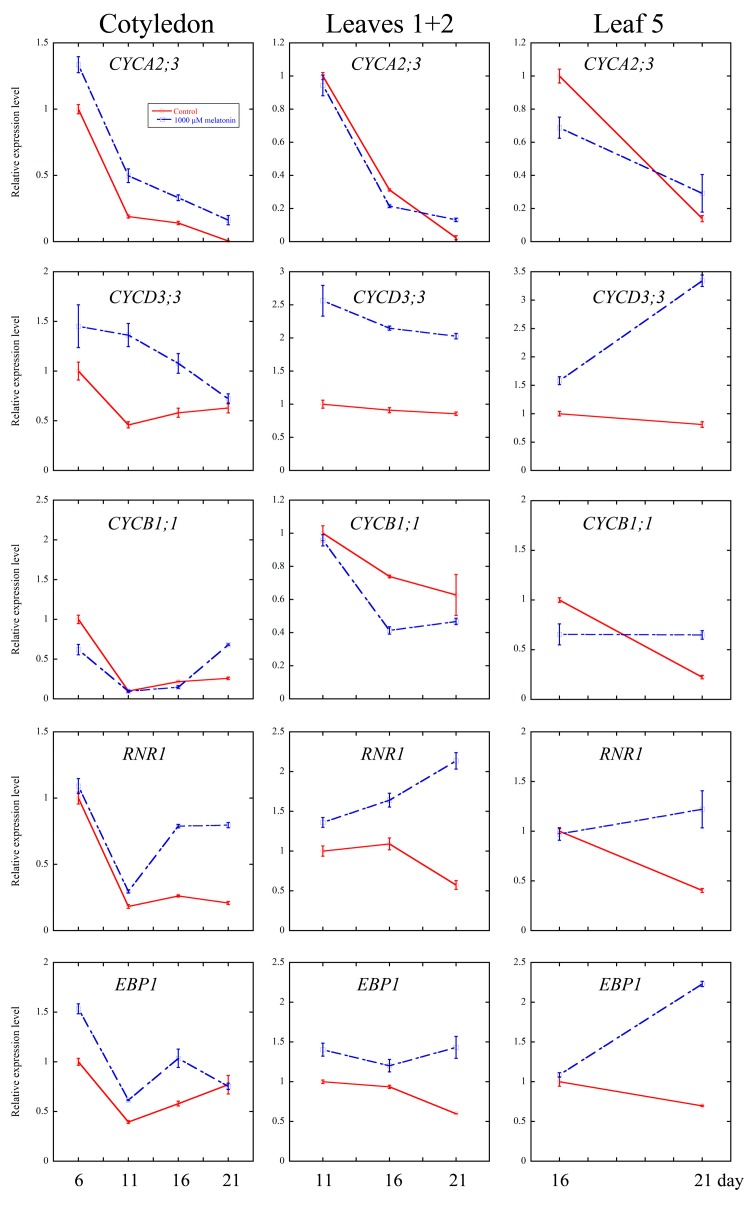

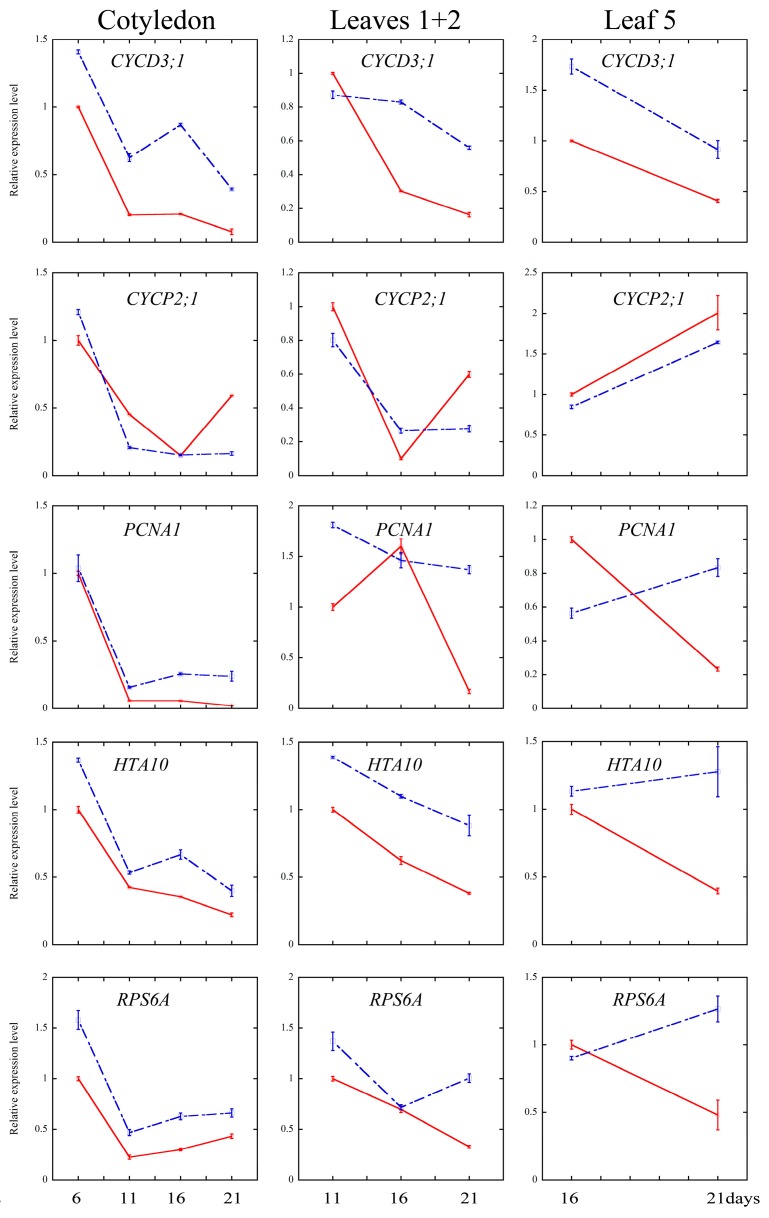
Quantitative real-time PCR (qRT-PCR) analysis of cell cycle and ribosomal related genes’ expression under control and 1000 μM melatonin treatment. Relative fold changes of the expression of *CYCA 2;3*, *CYCD 3;1*, *CYCD 3;3*, *CYCP 2;1*, *CYCB 1;1*, *PCNA1*, *RNR1*, *HTA10*, *EBP1* and *RPS6A* were quantified by real-time PCR, and the expression levels of the indicated genes in control leaves at six days were set to 1. Values represent mean ± SD.

Cell cycle is an essential process in organ growth, driven by a complex series of events and precisely regulated at the level of transcription especially [56]. Several core cell cycle genes have been widely identified [52,57,58,59,60]. To examine whether melatonin treatment affected the transcriptional levels of key genes of cell cycle, the quantitative real-time PCR analysis were performed on cotyledons (6-, 11-, 16- and 21-day treatment), leaves 1 + 2 (11-, 16- and 21-day treatment) and the fifth leaves (16- and 21-day treatment). *CYCA2;3*, *CYCD3;1* and *CYCD3;3* are key regulators of ploidy levels in *Arabidopsis* endoreduplication [60,61,62]. *CYCB1;1* and *CYCP2;1* are markers in G2-M phase activation in *Arabidopsis* [63,64]. *HTA10* (Histone H2A gene 10), *PCNA1* (Proliferating Cell Nuclear Antigen1) and *RNR1* (RiboNucleotide Reductase1) are S-phase gene markers [60,65]. As shown in Figure 5, the transcript levels of cyclins significantly increased in most of the development stage of all three tissues after the melatonin treatment, while those of *CYCP2;1* and *CYCB1;1* were down-regulated by the melatonin treatment. The S-phase key genes (*HTA10*, *PCNA1* and *RNR1*) showed significantly higher transcript levels at most of the time points after melatonin treatment.

Cell growth is consistent with the rate of protein synthesis [66]. Several ribosomal proteins play important role in morphological and developmental regulation of *Arabidopsis* [60,67,68]. Here, we found that the transcripts of *RPS6A* (Ribosomal Protein S6A) and *EBP1* (ERBB-3-Binding Protein1) were up-regulated by melatonin, indicating the involvement of protein synthesis in melatonin regulated cell growth.

## 3. Discussion

Melatonin, a natural product first found in plants less than 30 years ago, has been shown to play various functions in plants. In addition to its functions in regulating stress responses [6,33,35,69], melatonin was also proven to be involved in the regulation of plant growth [31]. We have found that high dose of melatonin inhibited the root growth in *Arabidopsis* seedlings and suppressed the floral transition in *Arabidopsis* in our previous studies [23,24]. Consistently, it has been reported that 1 mM melatonin treatment of four-week-old detached *Arabidopsis* leaves caused increased oxidative stress resistance [15]. However, whether melatonin also has an effect in regulating leaf growth is still unknown. In the present study, the effect of melatonin on the growth and development of *Arabidopsis* leaf was examined, and the underlying mechanism was also partially investigated.

### 3.1. Melatonin Represses Cell Proliferation and Cell Expansion in Arabidopsis Leaf Growth

In this study, we first tested the effect of melatonin on leaf growth of *Arabidopsis*. The results showed that low concentration of melatonin (1, 10 and 100 μM) had no effect on cotyledon size after treatment for six days (Figure 1B). Our preliminary experiments and previous studies indicated that low concentration of melatonin (10–50 μM) had little effect on the content of endogenous melatonin and the development of plant root [18,23]. However, higher concentration of melatonin (600 and 1000 μM) dramatically suppressed the size of *Arabidopsis* cotyledon in a dose-dependent manner (Figure 1B). After digging deeper into the effects of melatonin, we found that both palisade cell size and cell number were reduced by high concentrations of melatonin, consistent with its effects on cotyledon size (Figure 1A,C,D). It is generally believed that *Arabidopsis* cotyledon cells are highly differentiated in seeds already, and the growth of cotyledons is attributed to the cell expansion [70,71]. Further studies showed that the cell cycling of cotyledon palisade cells is reactivated after seeds imbibition, which relies on de novo sucrose synthesis [72]. Here, the kinetic analysis of cotyledons, the first pair of true leaves and the fifth leaves in control and 1000 μM melatonin treated seedlings showed coincident results (Figure 2), except the cell number of cotyledons in seedlings treated for 11, 16 and 21 days. Based on these results, melatonin may affect both cell proliferation and cell expansion in leaf growth.

The cell division rate and duration, as important parameters of cell proliferation, have significant impacts on the leaf size [51]. The decline of cell division rate of palisade cells in melatonin-treated seedlings (Figure 3) suggested that melatonin suppressed the cell number in *Arabidopsis* leaves by reduced cell division rate.

### 3.2. Melatonin Inhibites Endoreduplication during Leaf Growth

Endoreduplication, also known as endocycle, refers to the fact that cells undergo DNA replication without cell division, and is the mainly cause of cell ploidy change [73,74]. Moreover, the endocycle can lead to a larger nucleus and the enlargement of cell size in certain cells [75,76,77,78,79]. Small organ size in *bin4* is primarily caused by suppressed cell expansion associated with defects in endoreduplication [80]. Overexpression of *Arabidopsis thaliana* homeobox 12 (ATHB12) resulted in increased cell size, along with elevated ploidy [81]. Our results showed that 1000 μM melatonin caused significantly 2C fraction increase in cotyledons, the first pair of true leaves, and the fifth leaves, in accordance with the decrease of higher DNA content (Figure 4A–C). Similar findings were reported by Posmyk [82], that 100 μM pretreatment of melatonin in red cabbage seeds significantly inhibited DNA endoreduplication [82]. Moreover, the endoreduplication index (EI) of melatonin treated leaves was significantly lower than that of control leaves (Figure 4D). Therefore, melatonin affected the DNA ploidy and endoreduplication during the development of leaf. Jasmonate also negatively affects leaf size in *Arabidopsis*, and causes DNA content change by delaying the onset of endoreduplication [60]. It is well known that gibberellic acid (GA) promote plant growth through promoting the degradation of DELLAs, which were proven to perform the inhibitory activity of plant growth through reducing the rates of both cell proliferation and cell expansion [83]. In our recent studies, we found that melatonin-mediated protein stabilization of DELLAs in *Arabidopsis* [24]. Thus, DELLAs may also be involved in melatonin-mediated leaf development, and this needs to be further investigated.

### 3.3. Melatonin Regulates Cell Cycle Progression

On the one hand, it has been reported that both high concentrations (1 mM) and low concentrations (100 pM) of melatonin treatment can result in extensive transcriptional reprogramming in *Arabidopsis*, including the transcripts of various stress-related genes [15]. The extensive transcriptional reprogramming is consistent with the wide participation of melatonin in plant stress resistance, at least partially [11,12,13,14,15,16,17,18,19,20,21,22]. On the other hand, melatonin is also involved in several developmental processes of plants, including seed germination, shoot development, root growth and architecture, flowering and fruit ripening [23,24,25,26,27,28,29]. To our knowledge, this is the first study to report the involvement of melatonin in leaf development. In this study, we focused on the effect of melatonin on the development of leaf and the underlying mechanism. We found that high concentration of melatonin suppressed the cell proliferation and endoreduplication, and the expression of related key genes in leaf growth might contribute to this issue. Numerous genes were identified and shown to be involved in regulating cell cycle precisely on transcriptional level [52,57,58,59,60], and the mutations of some key genes of cell cycling lead to serious defects of leaf occurrence and development [65,84,85]. CYCA2;3, as a key regulator of DNA contents in *Arabidopsis*, negatively affects endocycles. Overexpression of *CYCA2;3* restrained endocycles of *Arabidopsis* leaves in a dose-dependent manner [62]. *CYCD3* subgroup of D-type cyclins *(CYCD)* family, composed of *CYCD3;1*, *CYCD3;2* and *CYCD3;3*, were shown to regulate the balance between cell division and cell expansion [61,86,87]. Overexpression of *CYCD3;1* dramatically repressed endoreduplication [61]. Triple mutant *cycd3;1-3* showed larger cells and initiated endoreduplication in petals that usually do not undergo endocycles [86]. In our study, the transcriptional level of *CYCA2;3* in cotyledons was elevated at all the time points after melatonin treatment, and in leaves 1 + 2 and leaf 5, the transcriptional level of *CYCA2;3* was also up-regulated after melatonin treatment for 21 days. Moreover, the expression levels of *CYCD3;1* and *CYCD3;3* were up-regulated by melatonin in all three tissues at most of the time points (Figure 5). Since *CYCA2;3*, *CYCD3;1*, *CYCD3;2*, and *CYCD3;3* are negative regulators of endoreduplication, the higher transcripts of these genes in the melatonin treated leaves might contribute to the inhibition of endoreduplication.

CYCP2;1 was proven to interact with CDKA;1, CDKB2;1 and CDKB2;2 with role in the transition of G2 to M phase during meristem activation, as evidenced by the significant G2 arrest of the *cycp2;1* mutants [65,88]. As shown in Figure 5, melatonin treatment down-regulated the expression level of *CYCP2;1* in cotyledons and leaves 1 + 2 at 11 and 21 days, and also in the fifth leaves at 16 and 21 days. At CYCB1;1 is a positive regulator and expressed only at the G2-M phase transition [89,90]. Our findings showed that melatonin treatment lead to lower expression of *CYCB1;1* in cotyledons and leaves. Similar results were found in the methyl jasmonate (MeJA) treated *Arabidopsis CYCB1;1*:*Dbox*-*GUS* seedlings, which showed down-regulation of *CYCB1;1* in the arrest of leaf development [54]. Reduction of transcriptional level of *CYCP2;1* and *CYCB1;1* indicated that melatonin negatively regulated the phase transition of G2 to M in *Arabidopsis* leaves, which is consistent with reduced cell number in the melatonin treated leaves (Figure 1, Figure 2 and Figure 3).

PCNA (Proliferating Cell Nuclear Antigen) is a key nuclear protein of eukaryotic dividing cells in the early S phase [91]. PCNA acts as a sliding platform loaded onto the DNA duplex, and involved in DNA synthesis, cell cycle regulation and DNA repair [92,93]. RNRs (Ribonucleotide reductases) are critical for DNA damage checkpoint pathways in yeast, mammals and higher plants like *Arabidopsis* [94,95]. The elevated expression level of *PCNA1* and the induced transcriptional level of *RNR1* in melatonin treated cotyledons and leaves were shown in this study. Histon H2A proteins consist of 13 members in *Arabidopsis* [96]. Several reports have shown that histone genes is coupled to the S phase, and emphasized their roles in DNA replication and cell proliferation [97,98,99,100]. Our experiments showed that the expression of *HTA10* was up-regulated by melatonin in all three tissues at all the time points. We also found that the transcript levels of some ribosomal genes, *EBP1* and *RPS6A*, were induced by melatonin treatment in all three tissues at most time points (Figure 5). Noir et al. have showed that keeping high expression levels of histones, ribosomal proteins and pre-RC components by MeJA could help the cells maintaining a potential stand-by mode [60]. The modulation of melatonin on these genes’ transcripts indicated that melatonin treated cells still hold their potency to recover from the exogenous treatment.

## 4. Experimental Section

### 4.1. Plant Materials and Growth Conditions

The *Arabidopsis thaliana* ecotype Columbia-0 (Col-0) was used as the WT plant. Seeds were sterilized with 70% (*v*/*v*) ethanol for 1 min and 1% sodium hypochlorite for 16 min. After washing with distilled water for 3–5 times, seeds were placed at 4 °C for 2 days to break dormancy. Stratified-seeds were then sown on half-strength Murashige and Skoog medium with 1% sucrose and 0.85% agar, and were transferred to a culture room under dark/light cycles of 8/16 h at the temperature of 22 °C for 24 h, to reduce the effect of melatonin treatment on seed germination. Seeds were then transferred to new plates with or without melatonin for another 6–21 day culture.

### 4.2. Leaf Measurements

*Arabidopsis* seeds germinating for one day were transferred to new 1/2 MS medium and medium containing different concentrations of melatonin. After another 6-day treatment, cotyledons of control and samples were first rinsed in bleach solution with 75% ethanol and 25% acetic acid for 2 h or overnight at 37 °C to remove chlorophyll, and then mounted in basic solution (7% sodium hydroxide in 70% ethanol) for 15 min at room temperature. After immersed in 60%, 40%, 20% and 10% ethanol for 20 min successively, the cotyledons were mounted with clearing solution (50 g of chloral hydrate, 15 mL of water and 10 mL of glycerol) on glass slides, and the palisade cells at the central region of a half leaf beside the mid-vein were captured with differential interference contrast (DIC) objective of Leica TCS SP8 (Leica, Mannheim, Germany) laser scanning confocal microscope to determine cell size and cell number per unit area [101]. Those slides were then photographed under a Leica M205 FA (Leica, Singapore) stereo microscope to determine the area of the cotyledons. The areas of both cotyledons and cells were measured by software Image J (Wayne Rasband, Bethesda, MD, USA, available online: http://rsbweb.nih.gov/ij/, version 1.47 g). The estimated total palisade cell numbers per cotyledon were calculated by cotyledon and cell areas. Cell division rate (d) was calculated from *N*_1_ = *N*_0_ 2*^dt^* [46]. *D* = (log_2_*N*_1_ − log_2_*N*_0_)/(*t*_1_ − *t*_0_). *N* and *t* represent the cell number and time (h) respectively. Results presented are average values of more than 10 seedlings per treatment from three independent experiments. Statistical analysis was conducted in KaleidaGraph 4.03 (Synergy Software, Reading, PA, USA).

### 4.3. Flow Cytometry Experiments and Ploidy Measurement

After one-day germination, seeds were transferred onto new 1/2 MS medium and medium supplemented with 1000 μM melatonin for another 21 days. Cotyledons, the first true leaves and the fifth leaves of control and melatonin treated samples were selected and rinsed in 1 mL cold Galbraith’s buffer (45 mM MgCl_2_, 20 mM MOPS, 30 mM Sodium Citrate, 0.1% Trtion X-100, pH 7.0 with 1 M NaOH) [102]. Cotyledons or leaves were cut by a blade irregularly and quickly, filtered through miracloth (22–25 μm, Millipore, Billerica, MA, USA). Filtrate was treated with RNase A with the final concentration of 50 μg/mL for 20 min, and then stained with Propidium Iodide (PI) (Sigma-Aldrich, St. Louis, MO, USA) with the final concentration of 50 μg/mL for 20 min at 4 °C. All the samples were measured by BD Accuri C6 Flow Cytometer. At least 10,000 nuclei isolated were used for ploidy measurement. Flow Cytometry Experiments were repeated at least three times from independent biological replicates. Statistical analysis was conducted in KaleidaGraph 4.03.

### 4.4 Quantitative Real-Time Pcr Analysis

One-day-old *Arabidopsis* seeds were transferred to new 1/2 MS medium and medium containing 1000μM melatonin. After another 6–21 day treatment, cotyledons (6-, 11-, 16-, and 21-day treatment), the first true leaves (11-, 16-, and 21-day treatment), and the fifth rosette leaf (16- and 21-day treatment) of control and samples were collected, and total RNA was isolated from leaves treated with TRIzol reagent (Invitrogen, Carlsbad, CA, USA). For cDNA synthesis, 2 μg of total RNA from different samples was used for reverse transcription with RevertAid First Strand cDNA Synthesis Kit (Thermo Scientific, Waltham, MA, USA) according to the manufacturer’s recommendations. To analyze the transcript levels of cell cycle-related genes in control and treated leaves, quantitative real-time PCR was performed with Roche LightCyler 96^®^ in a 20 μL reaction volume containing SYBR Green dye (FastStart Essential DNA Green Master, Roche, Mannheim, Germany). *AtPDF2* (protein phosphatase 2, AT1G13320) was chosen as an internal control [103]. Relative expression levels were estimated using the 2^−ΔΔ*C*t^ method [104]. All primers used in the study are listed in Supplementary Materials, Table S1.

## 5. Conclusions

In this study, we found that high concentration of melatonin caused smaller leaf size in *Arabidopsis* by reducing cell size and cell number, as confirmed by the kinetic analysis of leaf growth and flow cytometic analysis of leaf DNA ploidy. The results showed that melatonin suppressed the cell division rate and endoreduplication. Expression analysis of cell cycle genes and ribosomal genes suggested melatonin negatively regulated cell division and endocycle. In summary, this study provides a direct link between melatonin and leaf development, and emphasized the modulation of melatonin in retarded cell proliferation, endoreduplication and cell expansion. We also highlight the involvement of melatonin in the maintenance of a stand-by mode in *Arabidopsis* leaf cells.

## Supplementary Materials

Supplementary materials can be found at www.mdpi.com/1422-0067/18/5/991/s1.
