# Supplementary material for: High Concentration of Melatonin Regulates Leaf Development by Suppressing Cell Proliferation and Endoreduplication in Arabidopsis
Source: Int J Mol Sci. 2017 May 5;18(5):991. doi: 10.3390/ijms18050991 (PMC5454904; doi:10.3390/ijms18050991)
Supplement: Supplementary file 1 [file ijms-18-00991-s001.pdf]

Supplementary Information

Table S1. List of the primers used in this study.

**Primer name**	**Sequence (5' to 3')**
PDF2 F	TAACGTGGCCAAAATGATGC
PDF2 R	GTTCTCCACAACCGCTTGGT
CYCA2;3 F	TCTTGGGAGATCAGCTTCTACAGC
CYCA2;3 R	GGCATAGAGGCAGCACAGTAAAGG
CYCD3;1 F	TTCAAGTGGAGGAGACAAAGTATG
CYCD3;1 R	TCTACGAACGAAATTGGAGTAATGA
CYCD3;3 F	TGTCTGCTTCTGCTTCAGTGTCG
CYCD3;3 R	TGCTGCTCTTGCACTCTTCTCC
CYCP2;1 F	TAGATGACGTGCACTACAACAACG
CYCP2;1 R	CTGCATTGCTTACGCCTCCAAC
CYCB1;1 F	CACACTGGCTATTCTGAGACTC
CYCB1;1 R	TCTTCTTGTTGCTTCCATTGCTGA
PCNA1 F	CGGTGACATTGGAACCGCTAAC
PCNA1 R	TCACAATTGCATCTTCCGGCTTG
RNR1 F	TGACACAGCCATGCTCAAGGAG
RNR1 R	TTCCGCAGGCCAAACACTCTTC
HTA10 F	GGGTCGTATCGCTCGTTTCTTG
HTA10 R	TCCAGCCAATTCCAATACCTCAGC
EBP1 F	ATGCCATTCACTGCAAGGTCAC
EBP1 R	ACACACTCCACAAGTCCAAGCC
RPS6A F	GATCGACGATGACCAGAAACTACG
RPS6A R	GGGAAACCTTGCTTATCGCAAC
